# KSHV requires vCyclin to overcome replicative senescence in primary human lymphatic endothelial cells

**DOI:** 10.1371/journal.ppat.1008634

**Published:** 2020-06-18

**Authors:** Terri A. DiMaio, Daniel T. Vogt, Michael Lagunoff

**Affiliations:** Department of Microbiology, University of Washington, Seattle, Washington, United States of America; Oregon Health and Science University, UNITED STATES

## Abstract

Kaposi’s Sarcoma Herpesvirus (KSHV) is present in the main tumor cells of Kaposi’s Sarcoma (KS), the spindle cells, which are of endothelial origin. KSHV is also associated with two B-cell lymphomas, Primary Effusion Lymphoma (PEL) and Multicentric Castleman’s Disease. In KS and PEL, KSHV is primarily latent in the infected cells, expressing only a few genes. Although KSHV infection is required for KS and PEL, it is unclear how latent gene expression contributes to their formation. Proliferation of cancer cells occurs despite multiple checkpoints intended to prevent dysregulated cell growth. The first of these checkpoints, caused by shortening of telomeres, results in replicative senescence, where cells are metabolically active, but no longer divide. We found that human dermal lymphatic endothelial cells (LECs) are more susceptible to KSHV infection than their blood-specific endothelial cell counterparts and maintain KSHV latency to higher levels during passage. Importantly, KSHV infection of human LECs but not human BECs promotes their continued proliferation beyond this first checkpoint of replicative senescence. The latently expressed viral cyclin homolog is essential for KSHV-induced bypass of senescence in LECs. These data suggest that LECs may be an important reservoir for KSHV infection and may play a role during KS tumor development and that the viral cyclin is a critical oncogene for this process.

## Author summary

Kaposi’s Sarcoma (KS) is the most common tumor of AIDS patients world-wide and a significant tumor in sub-Saharan Africa. Kaposi’s Sarcoma herpesvirus (KSHV), the etiologic agent of KS, is found predominantly in the latent state in tumor cells where the virus expresses a limited number of genes. To clarify how KSHV latency induces KS tumor formation we sought to determine how KSHV infection alters blood and lymphatic endothelial cells. KSHV infection of human LECs promotes their proliferation beyond replicative senescence, the first step of oncogenesis. Using this system we are able to identify bona fide viral oncogenes in the context of viral infection of relevant human dermal endothelial cells. These studies provide insight into how KSHV induces KS tumor formation.

## Introduction

Kaposi’s Sarcoma herpesvirus (KSHV) is the etiological agent of Kaposi’s Sarcoma, a highly vascularized tumor made up of spindle cells. Spindle cells express specific markers of lymphatic endothelium including VEGF receptor-3 and prox-1, though some markers of blood endothelium are also expressed [1–5]. KSHV also infects B cells and is associated with two rare B-cell lymphomas, Primary Effusion Lymphoma (PEL) and Multicentric Castleman’s Disease [6–8]. Despite being necessary for KS and PEL formation, the mechanisms by which KSHV induces tumor formation in human cells are poorly understood.

The first step of tumor formation is overcoming replicative senescence. Primary cells have a limited lifespan due to shortening of their telomeres during cell division (reviewed in [9]). Shortening of the telomeres eventually leads to activation of the DNA damage response and the tumor suppressor proteins p53 and Rb. These proteins induce a permanent cell cycle arrest known as senescence. During senescence, cells no longer divide but are still metabolically active. In order for primary human cells to become transformed, both p53 and Rb are inactivated. PEL cells have been shown to inhibit p53 activity through latent expression of viral Interferon Regulatory Factor 3 (vIRF3). vIRF3 interacts with p53 to reduce its stability and DNA binding activity, thereby inhibiting its pro-apoptotic function [10, 11]. Furthermore, activation of p53 in PEL cells leads to reduced proliferation and apoptosis [12, 13]. This is also true for telomerase-immortalized HUVECs that have been transformed and maintain KSHV, however, expression of vIRF3 has not been detected during latent infection of these cells [14].

KS spindle cells predominantly express viral genes from the major latent locus, which is comprised of four genes and 12 viral miRNA loci. Several of these genes have been shown to regulate the cellular DNA damage response and tumor suppressor genes. For example, the latency associated nuclear antigen (LANA) has been shown to interact with both p53 and Rb, leading to inactivation of these proteins [15–17]. In addition, KSHV vCyclin, a homolog of cellular cyclins D and E, interacts with several CDKs, particularly CDK6, which results in a constitutively active cyclin/CDK complex and enhanced proliferation (reviewed in [18]). VCyclin expression is necessary for KSHV-transformed rat metanephric mesenchymal cells to override contact inhibition [19]. However, overexpression of vCyclin alone in endothelial cells induces oncogene-induced senescence [20]. Oncogene induced senescence is a different process than replicative senescence, the first due to activation of oncogenic signaling pathways and the second induced by shortening of telomeres. The KSHV vCyclin induced senescence can be relieved by co-expression of an additional viral gene in the latent locus, vFLIP [21]. The ratio of vCyclin and vFLIP seems to be important for regulating cell survival and proliferation since vCyclin expression is able to counteract senescence induced by vFLIP through activation of NF-κB [22]. Thus, KSHV manipulates the host cell through a variety of mechanisms to promote survival and proliferation but little is known about how KSHV alters the proliferation of human endothelial cells.

While KSHV infected spindle cells express markers of lymphatic endothelium, when KSHV infects blood ECs (BECs), it induces expression of lymphatic markers [23–25]. Therefore, it is unclear which endothelial cell type is relevant to the formation of spindle cells. Here we examine the differences between KSHV infection of neonatal blood and lymphatic endothelial cells. LECs are more susceptible to KSHV infection, and better able to maintain the viral episome. Importantly, KSHV infection promotes LECs, but not BECs, to proliferate beyond replicative senescence. However, KSHV is not sufficient for full immortalization of LECs. Proliferation of KSHV infected LECs past senescence is dependent on expression of the viral cyclin but not other members of the latent locus, such as vFLIP or the KSHV encoded miRNAs. These data suggest that KSHV preferentially infects lymphatic endothelial cells and can drive the first step towards tumor formation and that the viral cyclin is a key oncogene for KSHV induced proliferation past senescence and therefore likely plays a critical role in at least the first steps of KS tumorigenesis.

## Results

### LECs are more susceptible to KSHV infection than BECs

**Fig 1:**
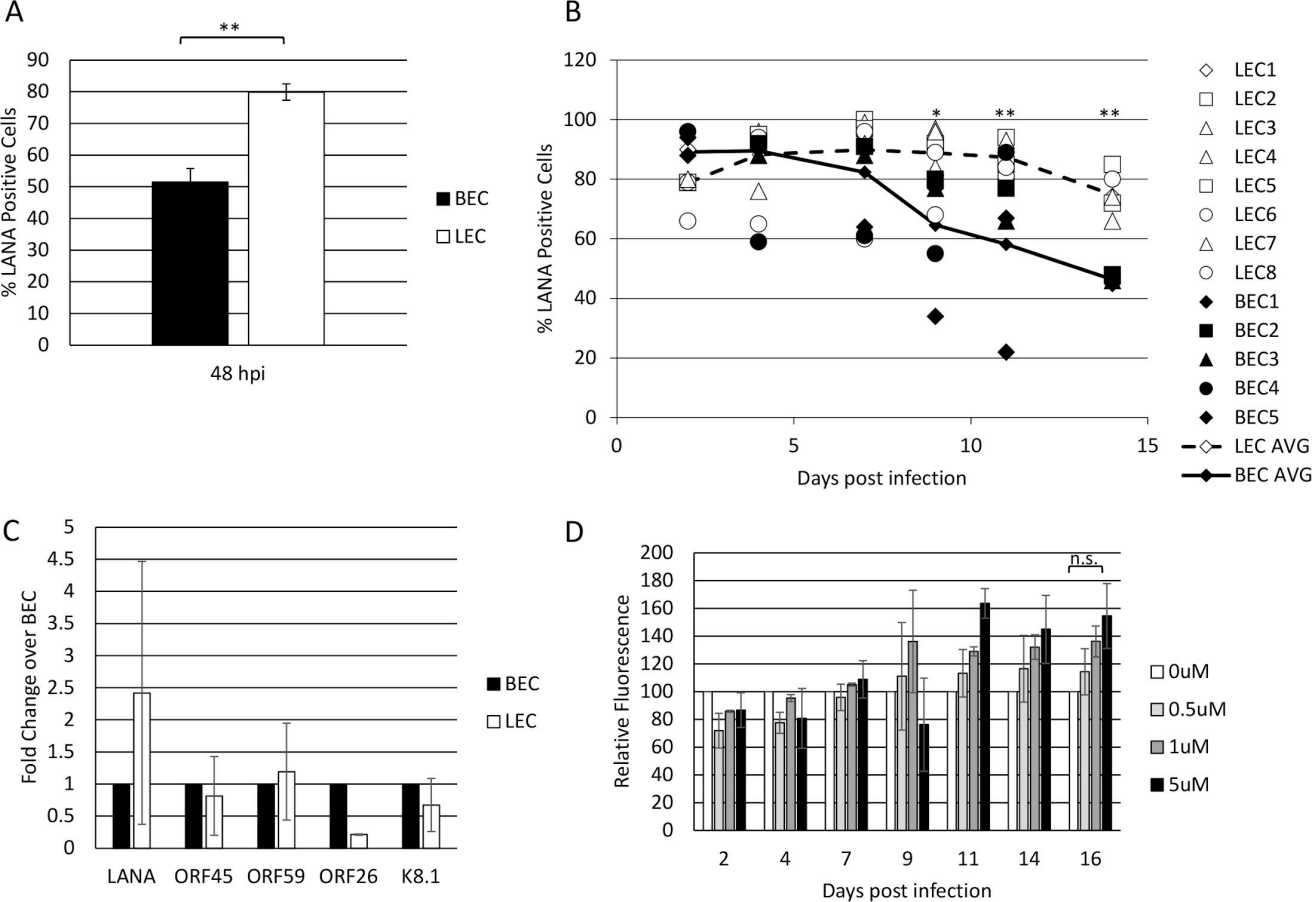
LECs are more susceptible to KSHV infection than BECs. A) Primary neonatal dermal blood and lymphatic endothelial cells were seeded at the same density and infected with KSHV at the same MOI. 48 hours post infection, cells were harvested, stained for immunofluorescence with an anti-LANA antibody, and the percentage of LANA+ cells was counted. B) BECs (closed markers) and LECs (open markers) were infected with KSHV and split 1:2 every 2–3 days, at which time the percentage of LANA+ cells were counted. Numbers indicate biological replicates of the experiment and the connected lines indicate the average of all replicates (BEC: solid line; LEC: dotted line). C) qRT-PCR of KSHV genes at 48 hpi of BECs and LECs. D) LECs were infected with WT BAC16KSHV and passaged in the presence of increasing concentrations of Ganciclovir. The relative intensity of GFP expression was measured using a Typhoon fluorescent imager and compared to untreated control cells using ImageJ software. Each of these experiments were performed at least twice.

Previously published data, as well as our own observations, suggest that LECs are more susceptible to KSHV infection than BECs [25, 26]. To further test this, we seeded 2x10^5 BECs or LECs (S1 Table) in 6-well dishes, allowed them to adhere, then infected with identical dilutions of KSHV. After 2 days, we harvested the cells and performed immunofluorescence for LANA expression to identify infected cells. A minimum of 200 cells per experiment were counted. The LECs were consistently infected to higher rates than the BECs with a range of 26 to 31 higher percentage of cells expressing LANA (Fig 1A). While the ability of KSHV to infect endothelial cells is dependent on a number of factors, LECs are consistently more highly infected than BECs.

### LECs maintain the KSHV viral episome

The KSHV genome is maintained as an episome in dividing cells, though this process is not robust, and the episome is lost over time in most cultured endothelial cells [27, 28]. To test whether the rate of loss is different between BECs and LECs, we infected cells with KSHV and harvested them every few days for immunofluorescence for LANA expression. Because LECs are more susceptible to KSHV infection, we added more virus to BECs to achieve similar initial infection rates. Fig 1B shows that, although they start at approximately 90% infection, BECs gradually lose the episome and by two weeks post infection fewer than 50% of cells exhibited punctate LANA staining. This was measured during five separate infections (BEC1 through BEC5 in Fig 1B) using three different isolations of cells from individual donors. In contrast, the initial infection rate of LECs was slightly lower in this set of experiments (approximately 80%) and increased until most of the cells were infected. The primary LECs maintained this high rate of infection for several weeks and over multiple passages and was seen in four different lots of cells from individual donors.

Previously published data suggests that KSHV-infected LECs exhibit a more lytic gene expression profile than BECs [26, 29]. Increased lytic replication could lead to reinfection of LECs with KSHV, allowing a higher percentage of cells to maintain latent gene expression. However, as shown in Fig 1C, in our hands, KSHV lytic gene expression is similar between BECs and LECs when the cells are passaged every two to three days after infection. Our previously published high-throughput RNA sequencing data showed no difference in viral gene expression between KSHV-infected BECs and LECs at 48 hours post infection [30]. In order to further confirm that the increased episome maintenance exhibited by KSHV-infected LECs is not due to increased viral replication, we passaged LECs infected with the recombinant KSHV expressing GFP in the presence of ganciclovir, which blocks KSHV genome replication [31]. Fig 1D shows that there is no significant decrease in the expression of GFP in cells passaged with ganciclovir as compared to those passaged in the absence of drug. There is a marginal, statistically insignificant, increase in GFP expression when lytic replication is inhibited. This may be due to an increased number of living cells, although further studies will be required to determine this. Regardless, this data suggests that lytic replication is not required for viral episome maintenance in neonatal LECs.

### KSHV infected LECs bypass replicative senescence

**Fig 2:**
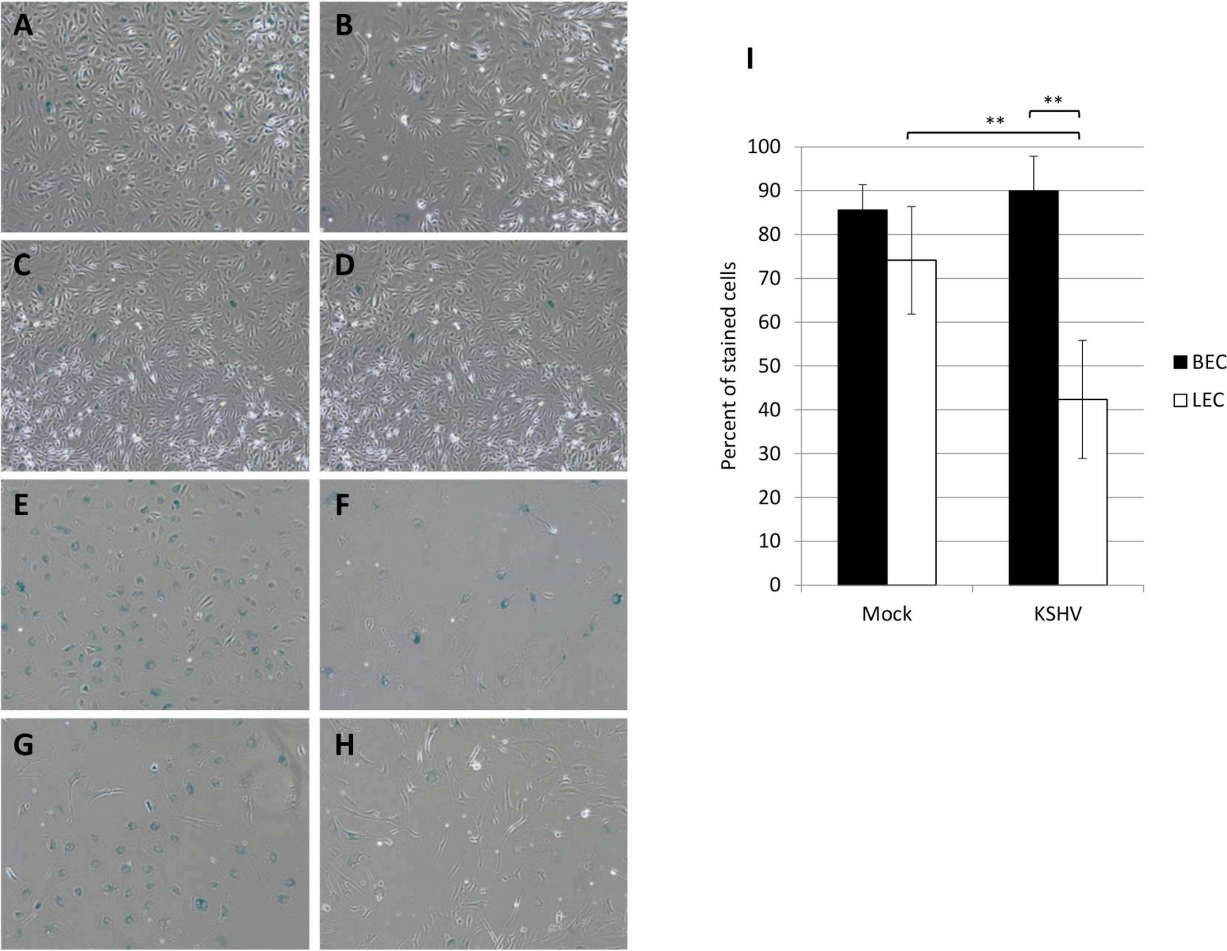
KSHV infected LECs bypass senescence. BECs (A, B, E, and F) and LECs (C, D, G, and H) were infected with KSHV (B, D, F, and H) or a no virus control (A, C, E, and G) at approximately passage 12 (A-D) and grown in the presence of 50μg/ml hygromycin until mock-infected cells began to show signs of senescence, approximately passage 15 (E-H). Cells were then fixed and stained for beta-galactosidase activity at low pH. Positive blue staining indicates senescence. This experiment was performed four times with at least 2 different isolates of each cell type. Representative images are shown. I) Quantitation of the percentage of cells staining positive at late passage in five images per condition.

Primary cells have a limited lifespan due to telomere shortening during DNA replication. In culture, this amounts to approximately 40–60 population doublings before cells begin to senesce. During senescence the cells remain metabolically active, but no longer divide. To determine if KSHV infection enables cells to proliferate past senescence, we infected both BECs and LECs with KSHV at approximately passage 12 and continued to passage them 1:2 until the uninfected cells began to senesce as determined by flattened shape and lack of proliferation. Cells were passaged under selection with hygromycin in order to maintain high levels of infection in the BECs. Once the uninfected cells appeared senescent, the cells were fixed and stained for beta-galactosidase activity at low pH and counted to quantify the senescence phenotype identified by microscopy. At early passage, both mock- and KSHV-infected BECs and LECs were largely negative for beta-galactosidase staining, indicating growing cells (Fig 2A–2D). In contrast, at the later passage, the cells in the mock and KSHV infected BECs and the mock infected LECs all had a flattened appearance indicative of senescence (Fig 2E–2G). However, the KSHV infected LECs had a normal cell appearance (Fig 2H). To confirm and quantify this effect the percentage of cells staining blue in the beta-galactosidase assay were counted. 80% of both mock- and KSHV-BECs stained positive for beta-galactosidase activity, a marker of senescence (Fig 2I). The majority of mock-infected LECs also stained positive. In contrast, fewer than 50% of KSHV-infected LECs exhibited beta-galactosidase staining, suggesting they are not senescent and are continuing to proliferate (Fig 2I).

**Fig 3:**
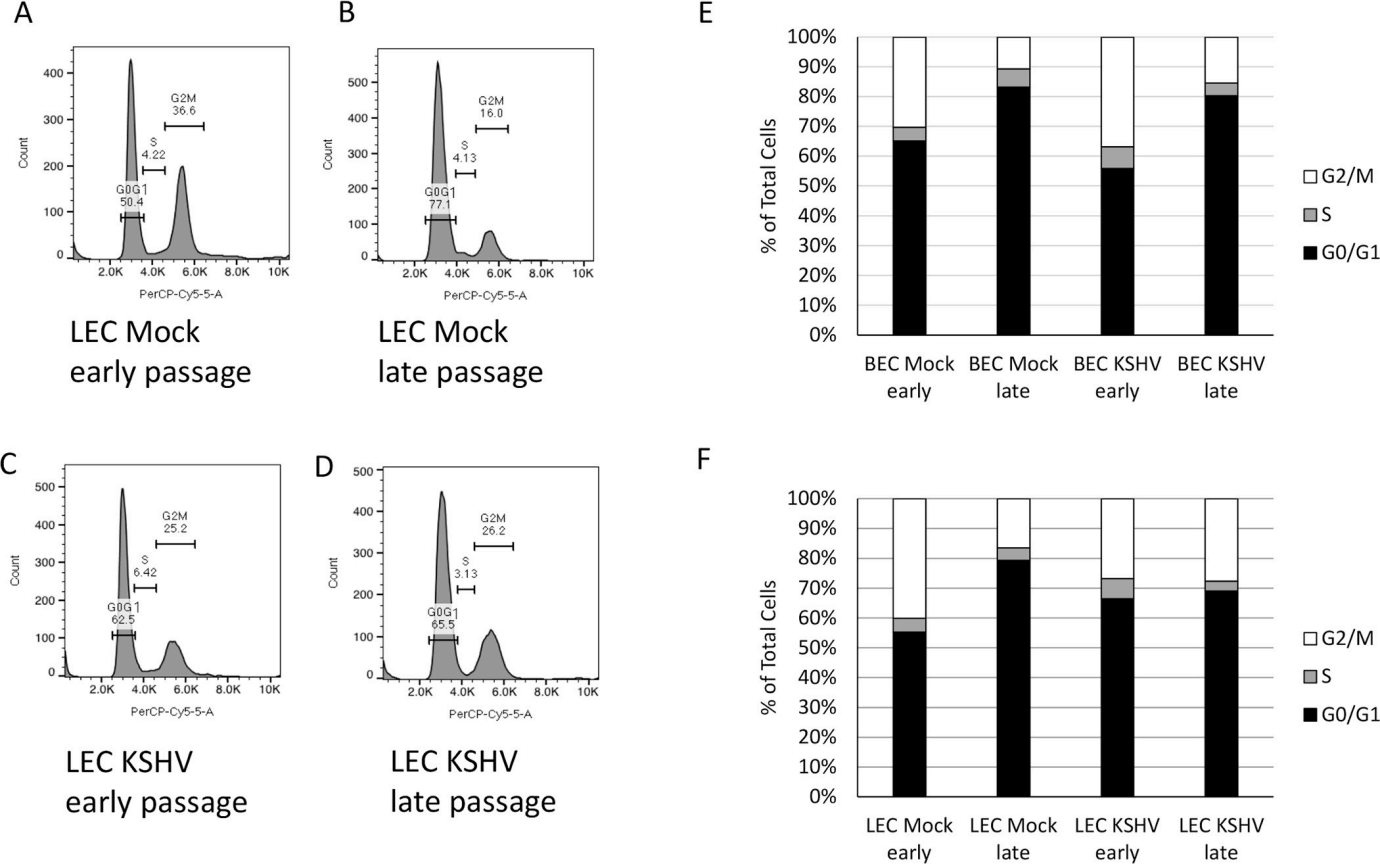
Senescent cells arrest in G0/G1. Early and late passage mock and KSHV-infected BECs and LECs were harvested, fixed, and stained with propidium iodide. Cell cycle was analyzed by flow cytometry. A-D) Representative histograms from early passage (A and C) and late passage (B and D) mock (A and B) and KSHV-infected (C and D) LECs showing percentage of cells in each phase of the cell cycle. E) Quantification of the percentage of cells in each phase of the cell cycle of early and late passage mock- and KSHV-infected BECs from a representative experiment. F) Quantification of the percentage of cells in each phase of the cell cycle of early and late passage mock- and KSHV-infected LECs from a representative experiment. This experiment was performed three times with similar results.

To further confirm that KSHV-infected cells had bypassed replicative senescence, we analyzed the cell cycle using propidium iodide and flow cytometry. Mock and KSHV-infected BECs and LECs were harvested at both early and late passages, fixed with methanol and stained with propidium iodide. Analysis of the cells by flow cytometry revealed that compared to early passage cells (Fig 3A and 3C), late passage mock-infected cells had a higher percentage of cells arrested in the G0/G1 phase of the cell cycle. The changes in the percent of cells in each population is consistent with a senescent phenotype described in the literature [22, 32]. In contrast, late passage KSHV-infected LECs (Fig 3D) maintained around 26% of cells in G2/M phase, suggesting continued proliferation. Fig 3E and 3F show quantification of a representative experiment comparing the percentage of BECs and LECs in each phase of the cell cycle. In Fig 3E, both mock- and KSHV-infected BECs become arrested in the G0/G1 phase of the cell cycle. Additionally, the mock-infected LECs become arrested in G0/G1 at late passages (Fig 3F). In contrast, KSHV-infected LECs had similar distribution of cells at both early and late passages (Fig 3F). Taken together, these data suggest that KSHV promotes the proliferation of LECs, but not BECs, beyond replicative senescence.

**Fig 4:**
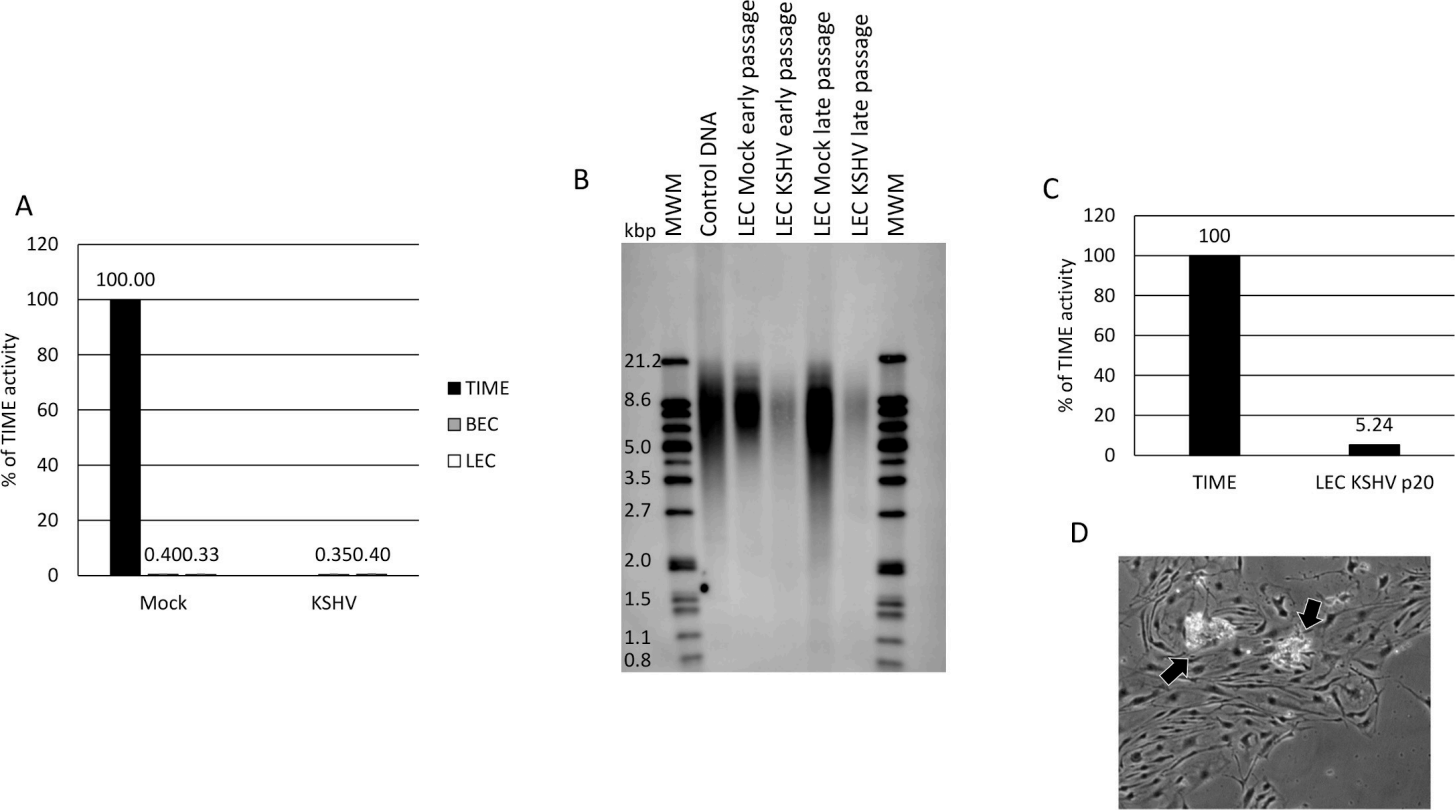
KSHV does not induce telomerase activity in LECs. A) BECs (Gray bars) and LECs (White bars) were infected with KSHV or no virus control and cells were harvested at 48 hpi. Cell lysates were subjected to a PCR-based TRAP assay for telomerase activity. TIME cells were used as a positive control (Black bar). This experiment was performed 3 times with similar results. B) Southern blot of total DNA from early and late passage mock- and KSHV-infected LECs probed with a telomerase-specific probe. This experiment was performed twice with similar results. C) KSHV-infected LECs were harvested at passage 20 and subjected to a PCR-based TRAP assay for telomerase activity. TIME cells were used as a positive control. D) Phase-contrast image of KSHV-infected LECs at passage >20. Arrows indicate areas where cells are dying, likely due to crisis.

We next asked whether KSHV infection activates telomerase activity. To do this, cells were mock- or KSHV-infected for 48 hours, then harvested and subjected to a PCR-based TRAP assay. As a positive control, we used TERT-immortalized microvascular endothelial (TIME) cells. Fig 4A shows that, unlike TIME cells, primary LECs and BECs have no detectable telomerase activity. In addition, KSHV does not increase telomerase activity in either BECs or LECs during early passage (Fig 4A).

We next analyzed the average telomere length using telomerase restriction fragment digestion. Total genomic DNA was isolated from early and late passage mock- and KSHV-infected LECs and subjected to digestion by Hinf1 and Rsa1 restriction enzymes. The DNA fragments were then run on a 0.8% agarose gel and transferred via southern blot to a positively charged nylon membrane and probed using a telomere-specific probe. Fig 4B shows that late passage mock-infected LECs had shorter telomere lengths compared to early passage LECs as indicated by the faster mobility smear on the gel (compare lane 5 to lane 3). Interestingly, late passage KSHV-infected LECs had telomere lengths that more closely resembled early passage cells (compare lane 6 to lanes 3 and 4; even with darker exposure, there only slight evidence of shortened telomeres in the KSHV-infected late-passage LECs (S1 Fig). Because of this, we measured the activity of telomerase in KSHV-infected LECs at passage 20, after all other cells had undergone senescence. Fig 4C shows that the telomerase activity of KSHV-infected LECs at passage 20 is approximately 5% of that of TIME cells. This is approximately 10-20-fold higher than that observed in early passage mock- and KSHV-infected cells, suggesting that there may be a slight induction of telomerase activity in KSHV infected LECs at late passages. However, it does not appear that this level of telomerase activity is sufficient to induce immortalization of primary cells. KSHV-infected LECs begin to die off at low levels after approximately passage 20 and cannot be passaged indefinitely (Fig 4D); about 3–5 passages post-senescence of the uninfected cells; [33–34]. This cell death is not synchronous, however, and suggests the cells are succumbing to crisis. Because death during crisis is asynchronous, there is not a reliable measure to quantify the level of cells undergoing crisis. However, in multiple attempts we were unable to grow the infected LECs beyond passage 20–22 despite the fact that they never adopted a senescent phenotype nor had high percentages stain positive for beta-galactosidase. This suggests that KSHV promotes LECs to bypass senescence but is insufficient to drive the cells past crisis to full immortalization.

### KSHV vCyclin is required for bypassing replicative senescence

**Fig 5:**
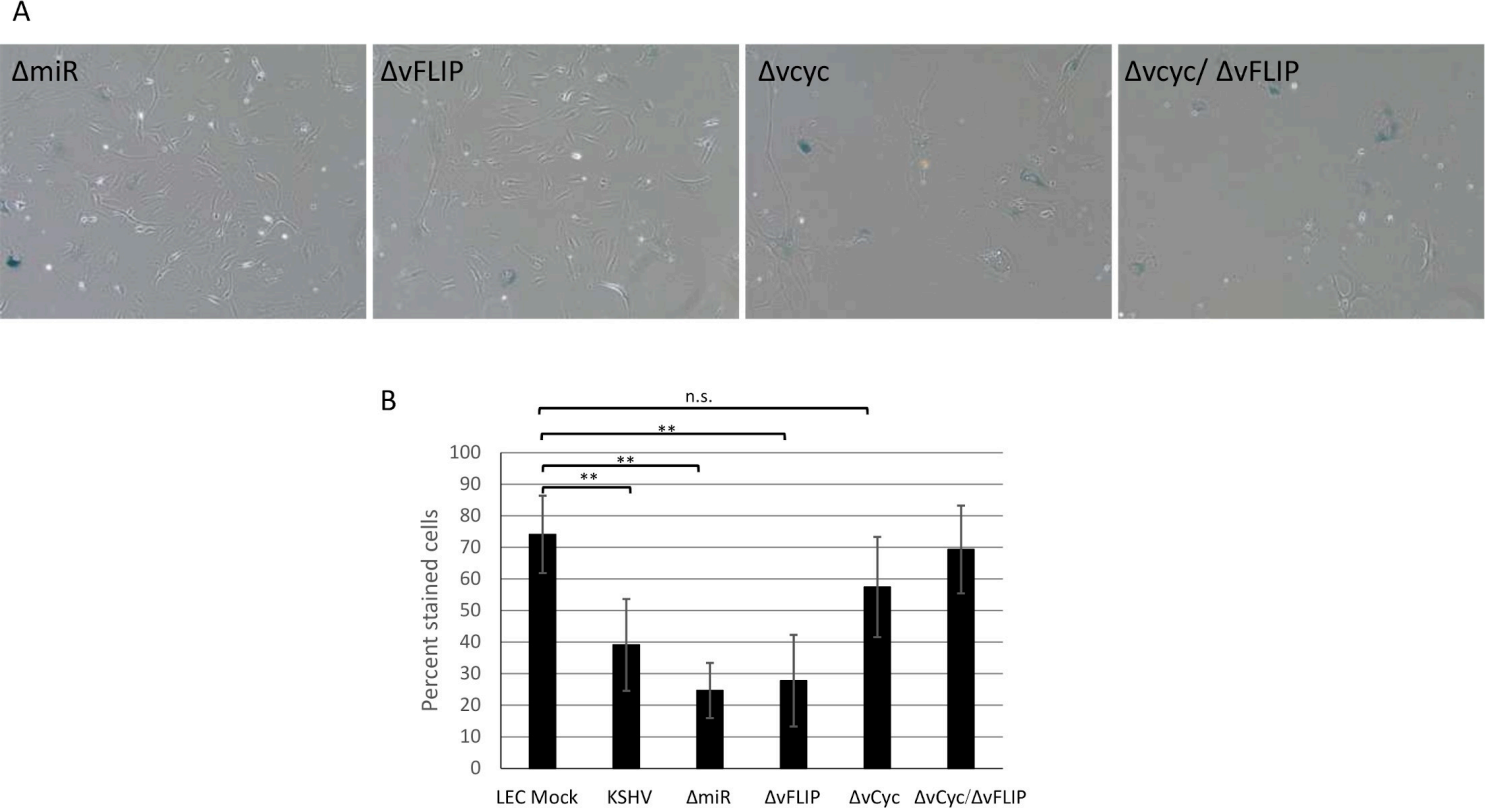
KSHV vCyclin is required for bypassing senescence. LECs were infected with mutant KSHV lacking individual latent genes. Cells were grown in the presence of 50μg/ml hygromycin until mock-infected cells began to show signs of senescence, approximately passage 15. Cells were then fixed and stained for beta-galactosidase activity at pH 6.0. A) Phase-contrast images of stained cells. These experiments were performed 4 times. Representative images are shown. B) Quantitation of the percentage of cells staining positive in five images per condition.

To determine which latent gene or genes are required for KSHV-infected LECs to bypass senescence, we obtained KSHV BAC16 constructs lacking either vCyclin, vFLIP, the viral microRNAs, or both vCyclin and vFLIP (provided by the Renne lab; [35–36]. We infected LECs with these viral constructs and grew the infected cells in the presence of hygromycin, to maintain high level infection, until mock-infected cells started to show morphologic signs of senescence, after which they were fixed and stained for beta-galactosidase activity at low pH. The cells infected with the ΔmiR or the ΔvFLIP viral mutants maintained the normal cell phenotype at late passage while cells infected with a virus lacking vCyclin or a double deletion of vCyclin and vFLIP became flattened and spread out, indicative of senescence (Fig 5A). As before, we confirmed and quantified these results with beta-galactosidase staining. Fewer than 40% of the LECs infected with either the ΔmiR or the ΔvFLIP viral mutants stained positive for beta-galactosidase activity, indicating that these genes are not required for LECs to bypass senescence (Fig 5B). In contrast, the majority of cells infected with virus lacking vCyclin senesced at the same passage as uninfected LECs despite high level infection. These data demonstrate that vCyclin is required for KSHV-induced proliferation past senescence.

## Discussion

KSHV infection is required for development of KS, as well as several B-cell tumors, however the mechanisms through which KSHV promotes tumor formation in human cells are not well understood. To better understand the origin of KS tumor cells and the viral mechanism of oncogenesis in human cells we sought to determine the role of KSHV in overcoming the first block to cellular oncogenesis, replicative senescence, in different endothelial cell types. KSHV infection of human dermal endothelial cells does not lead to their transformation, though it does promote cell survival [37]. Additionally, although KSHV infects both blood and lymphatic ECs in culture and KS spindle cells express markers of both BECs and LECs [1–5], it is not clear whether BECs or LECs are more important for KS development. Here we show that human dermal neonatal LECs are more susceptible to KSHV infection than BECs, and KSHV episome maintenance is more robust in LECs. Importantly, KSHV-infection of LECs, but not BECs, promotes their proliferation beyond normal replicative senescence, the first step in cell immortalization. These data suggest that lymphatic endothelial cells may be an important reservoir for latent viral maintenance and thus, may contribute to KS tumorigenesis.

Transformation of primary cells is a multistep process, the first of which is bypassing replicative senescence. Replicative senescence occurs due to shortening of telomeres during cell division. Shortened telomeres activate DNA damage checkpoint pathways, leading to activation of p53 and Rb. Our data suggest that KSHV infection allows LECs but not BECs to bypass these checkpoints. The mechanism through which KSHV does this in LECs remains unclear. Several studies have shown that KSHV latent genes are able to inhibit p53 and Rb activity, however whether this occurs in LECs and not BECs has not been carefully studied. In most PEL cell lines it appears that p53 signaling is functional [38]. More data will be needed to determine this in human endothelial cells. After senescence has been bypassed, telomeres continue to shorten with continued proliferation. A second checkpoint, termed crisis, happens when DNA damage due to lack of telomeres becomes widespread and cells begin to undergo apoptosis due to chromosome instability. In order for cells to become immortalized and transformed, they must upregulate telomerase activity to prevent crisis. While there may be some induction of telomerase activity in late passage LECs, KSHV does not strongly induce telomerase activity, which is consistent with the inability of KSHV to fully immortalize primary cells.

High-throughput sequencing of the transcriptomes of LECs and BECs upon infection with KSHV indicate there are a number of differences in the host response to KSHV infection in these cells (S2 Fig). This gene expression data is available at the NCBI Gene Expression Omnibus through GEO Series accession number GSE54416 (http://www.ncbi.nlm.nih.gov/geo/query/acc.cgi?acc=GSE54416). As we could not identify significant differences in viral gene expression between LECs and BECs, the highly divergent host gene expression of the different cell types upon infection is likely to underlie the differences in the ability of KSHV to allow differential growth properties in the two cell types. However, the LEC-specific gene expression pathway(s) essential for evading replicative senescence in LECs and not BECs remains to be determined. Work is underway to delineate which LEC specific host pathway is required for KSHV to allow cells to overcome senescence.

Using this cell senescence system we are able to identify bona fide viral oncogenes in a human cell type relevant to KS spindle cells. To identify KSHV latent genes necessary for preventing senescence in the context of viral infection we used a number of KSHV recombinants deleted for specific latent genes or miRNAs. These studies revealed that the viral cyclin homolog is essential for KSHV induced prevention of senescence in LECs. While the vCyclin gene is required for KSHV-induced bypass of senescence in LECs, its role in this process remains to be determined. In order to gain further insight into the role of v-Cyclin in influencing cellular gene expression during infection, we performed high-throughput RNA sequencing on LECs infected with either wild type BAC16-KSHV or the BAC16-ΔvCyclin mutant. This data is available at GEO Series accession number GSE136654 (https://www.ncbi.nlm.nih.gov/geo/query/acc.cgi?acc=GSE136654). Gene set enrichment analysis of these data sets suggests that genes involved in the JAK/STAT signaling pathway are enriched during wild type KSHV infection of LECs compared to LECs infected with KSHV-ΔvCyclin (S2 Table). We have previously shown that STAT3 signaling is constitutively activated during latent KSHV infection [39]. STAT3 signaling has been shown to repress p53 expression and activity, thus dysregulation of this pathway may play an important role in regulating cell proliferation and senescence [40]. Importantly, genes involved in the p53 pathway were found to be enriched during KSHV-ΔvCyclin infection of LECs compared to wild type KSHV (S3 Table). However, whether JAK/STAT signaling, and the p53 pathway are differentially regulated during KSHV infection of BECs or LECs still needs to be examined.

While many studies have examined KSHV induced changes to endothelial cells and many of these changes could be relevant to KS tumor formation, there is scant knowledge about KSHV induction of human endothelial cells to grow past senescence and become immortal. These studies provide some insight into how actual viral infection with KSHV overcomes the first checkpoint of unlimited cell proliferation, replicative senescence, and identifies a latently expressed viral gene essential for driving proliferation past senescence. Future work will further characterize how vCyclin is able to overcome replicative senescence in lymphatic endothelial cells.

## Materials and methods

### Cells

Primary neonatal human dermal microvascular blood and lymphatic endothelial cells were obtained from Lonza, generally at passage 3 (Lonza; LEC product #CC-2812; BEC product #CC-2813) and their identity was confirmed by PCR with primers that recognize BEC and LEC specific genes (S1 Table). Primary endothelial cells and TIME (Tert-Immortalized Microvascular Endothelial; 27) cells were maintained as monolayer cultures in EBM-2 medium (Lonza) supplemented with 5% fetal bovine serum, vascular endothelial growth factor, basic fibroblast growth factor, insulin-like growth factor 3, epidermal growth factor, and hydrocortisone (EGM-2 media). All experiments were carried out on cells between passage 7 and 12, unless otherwise specified.

### Viruses and infection

KSHV inoculum was obtained from BCBL-1 cells (5 x 10^5^ cells/ml) induced with 20 ng of TPA (12-*O*-tetradecanoylphorbol-13-acetate; Sigma)/ml as described previously [27]. After 5 days, cells were pelleted, and the supernatant was run through a 0.45-μm-pore-size filter (Whatman). Virions were pelleted at 30,000xg for 2 h in a JA-14 rotor, Avanti-J-25 centrifuge (Beckman Coulter). The viral pellet was resuspended in EBM-2 without supplements.

The recombinant KSHV BAC16 originally made in the Jung lab [41] was obtained from the Renne lab (see acknowledgements). The KSHV BAC16 mutant viruses were obtained from the Renne lab and are described elsewhere [35–36]. BAC16 and mutant viruses were passaged in iSLK cells as described [35]. In addition, viruses were confirmed for their mutation by PCR and sequencing. Lytic replication was induced by adding 1 μg/mL of doxycycline and 1 mM of sodium butyrate. Virus was harvested from the supernatant 4 days post induction as described above.

KSHV infections of primary hDMVEC were performed in serum-free EBM-2 supplemented with 8ug/ml polybrene for 3 hours, after which the medium was replaced with complete EGM-2. Mock infections were performed identically except that concentrated virus was omitted from the inoculum.

### Immunofluorescence

Mock- or KSHV-infected cells were seeded on LabTek Permanox four-well chamber slides (Fisher Scientific) and fixed with 4% (wt/vol) paraformaldehyde in phosphate-buffered saline. Immunofluorescence was performed as described previously [27]. Briefly, cells were incubated in Tris-Buffered Saline (20 mM Tris, 150 mM NaCl, pH 7.6; TBS) containing 1% normal goat serum followed by incubation with primary antisera at a dilution of 1:1,000 (rabbit anti-LANA) diluted in TBS containing 1% BSA for 1 hour. Cells were then incubated with fluor-conjugated secondary antibodies (Molecular Probes/Invitrogen) for 1 hour. Cells were mounted in medium containing DAPI (4',6'-diamidino-2-phenylindole) before being viewed under a Nikon Eclipse E400 microscope.

### RNA isolation and quantitative RT-PCR

Total RNA was isolated from mock- and KSHV-infected primary BECs and LECs using the RNeasy Plus Minikit (Qiagen). One hundred or 500 ng of total RNA was used in a SuperScript III, Platinum SYBR green, one-step, quantitative reverse transcription PCR (RT-PCR; Invitrogen) according to manufacturer's protocols with the primers for either GAPDH (glyceraldehyde-3-phosphate dehydrogenase) (forward, 5'-AAG GTG AAG GTC GGA GTC AAC G-3'; reverse, 5'-TGG AAG ATG GTG ATG GGA TTT C-3') or the viral gene LANA (forward 5’- TTG CCA CCC ACG CAG TCT-3’ reverse 5’- GGA CGC ATA GGT GTT GAA GAG TCT-3’), ORF45 (forward 5′-CAA CTC TCC GGA CGT GAA CA-3 reverse 5′-GGA GAT TGG GTT GGG AGG TG-3′), ORF59 (forward 5′-GAC AGC GTC TCG CTG ACA GA-3′ reverse 5′-CAC ACG CGT GAG CTA TTC GG-3′), ORF26 (forward 5′-AGC CGA AAG GAT TCC ACC ATT-3′ reverse 5′-CCG TGT TGT CTA CGT CCA GA-3′), or K8.1 (forward 5′-AAA GCG TCC AGG CCA CCA CAG-3 reverse 3′-GGC AGA AAA TGG CAC ACG GTT-5′). Relative abundances of viral mRNA were normalized by the delta threshold cycle method to the abundance of GAPDH. Error bars reflect standard errors of the means (four experiments).

### Senescence assay

Senescence-associated beta-galactosidase activity was measured using a kit from Cell Signaling (#9860). Briefly, cells were passaged in normal growth media and harvested at either early passage (approximately passage 12) or once the uninfected cells showed signs of visible senescence. Cells were then washed with PBS and fixed for 15 minutes at RT. Plates were washed again with PBS and beta-galactosidase staining solution was added to each well. Plates were sealed with parafilm and incubated overnight at 37°C. Pictures were taken using a BZ-X fluorescent microscope (Keyence).

### Flow cytometry

Monolayers of cells were washed once with PBS containing 0.04% EDTA and incubated with cell dissociation solution (Sigma) to remove cells from plates. Cells were fixed with 100% cold methanol, washed twice with PBS, then stained with PBS containing 100ug/ml RNaseA and 50ug/ml propidium iodide. Cells were then analyzed on a FACS Canto II flow cytometer (Becton-Dickinson, Franklin Lakes, NJ). Data was analyzed using FloJo flow cytometry analysis software (Tree Star, Inc.; Ashland, OR).

### TRAP assay

Telomerase activity was measured using a telomeric repeat amplification protocol (TRAP) assay as described in [42]. Briefly, cell pellets were resuspended in 10mM Tris-HCl (pH 7.5) containing 1mM MgCl_2_, 1mM EGTA, 0.1mM phenylmethylsulfonyl fluoride, 5mM β-mercaptoethanol, 0.5% CHAPS and 10% glycerol (1000cells/μl), incubated on ice for 30 minutes and then centrifuged for 30 mins at 10,000 x g at 4°C. Supernatant was placed in a fresh tube, flash frozen, and stored at -80°C. The SYBR Green RQ-TRAP assay was conducted with cell extracts (10,000 cells), 0.1μg of telomerase primer TS (5’ -AATCCGTCGAGCAGAGTT- 3’), and 0.05μg of anchored return primer ACX (5’–GCGCGG[CTTACC]_3_CTAACC- 3’), in 25μl with SYBR Green PCR Master Mix (Bio-Rad SSOAdvanced). Using the Bio-Rad CFX Connect thermal cycler, samples were incubated for 20min at 25°C and amplified in 35 PCR cycles with 30s at 95°C and 90s at 60°C. The threshold cycle values (C_t_) were compared with standard curves generated from serial dilutions of TERT-immortalized human microvascular endothelial (TIME) cell extracts (10,000, 1000, 100, 10, 1 cell).

### Telomere restriction fragment analysis

Southern blotting was performed using the TeloTAGGG kit (Roche Diagnostics, Indianapolis, IN, USA). Briefly, restriction digested genomic DNA (1–1.5 μg) was loaded onto a 10 cm 0.8% ultra pure agarose gel and run for 2 to 4 hours at 75v. The gel was incubated in hydrocholoric acid solution, denatured and neutralized before transferring overnight to a positively charged nylon membrane with 20× SSC. The DNA was fixed by exposing the membrane to UV irradiation. The membrane was pre-hybridized for 60 minutes at 42°C in DIG Easy Hyb solution before hybridization for 3 hours at 42°C with a telomere probe (5 μl probe in 5 ml pre-warmed DIG Easy Hyb). The membrane was washed and then blocked and incubated with anti-DIG antibody; after another round of washing the signal was detected using the supplied substrate solution and imaged using an Odyssey CLx imaging system (LiCor Biosciences).

### Statistics

Statistical differences between groups were evaluated with either Student’s *t* test (two-tailed) or one-way analysis of variance (ANOVA). Mean ± SD is shown and a *p*-value of ≤0.05 was considered significant and is indicated by asterisk; *p*-value of ≤0.01 is indicated by a double asterisk.

## Supporting information

S1 Fig KSHV-infected LECs lack shortened telomeres at late passage. Southern blot of total DNA from early and late passage mock- and KSHV-infected LECs probed with a telomerase-specific probe. A darker exposure of Fig 4B.
(TIF)

S2 Fig Differential expression of genes regulated by KSHV in BECs and LECs. (A) Venn diagram showing genes that are upregulated by KSHV in BECs (dark circle) and LECs (light circle). (B) Venn diagram showing genes that are downregulated by KSHV in BECs (dark circle) and LECs (light circle).
(TIF)

S1 Table Expression data for blood and lymphatic endothelial cell markers in different cell isolates. (DOCX)

S2 Table Pathways upregulated by WT KSHV but not ΔvCyclin. (DOCX)

S3 Table Pathways downregulated by WT KSHV but not ΔvCyclin. (DOCX)

S1 Text Supplemental materials and methods. (DOCX)
