# Supplementary figures and images for: KSHV requires vCyclin to overcome replicative senescence in primary human lymphatic endothelial cells
Source: PLoS Pathog. 2020 Jun 18;16(6):e1008634. doi: 10.1371/journal.ppat.1008634 (PMC7326280; doi:10.1371/journal.ppat.1008634)

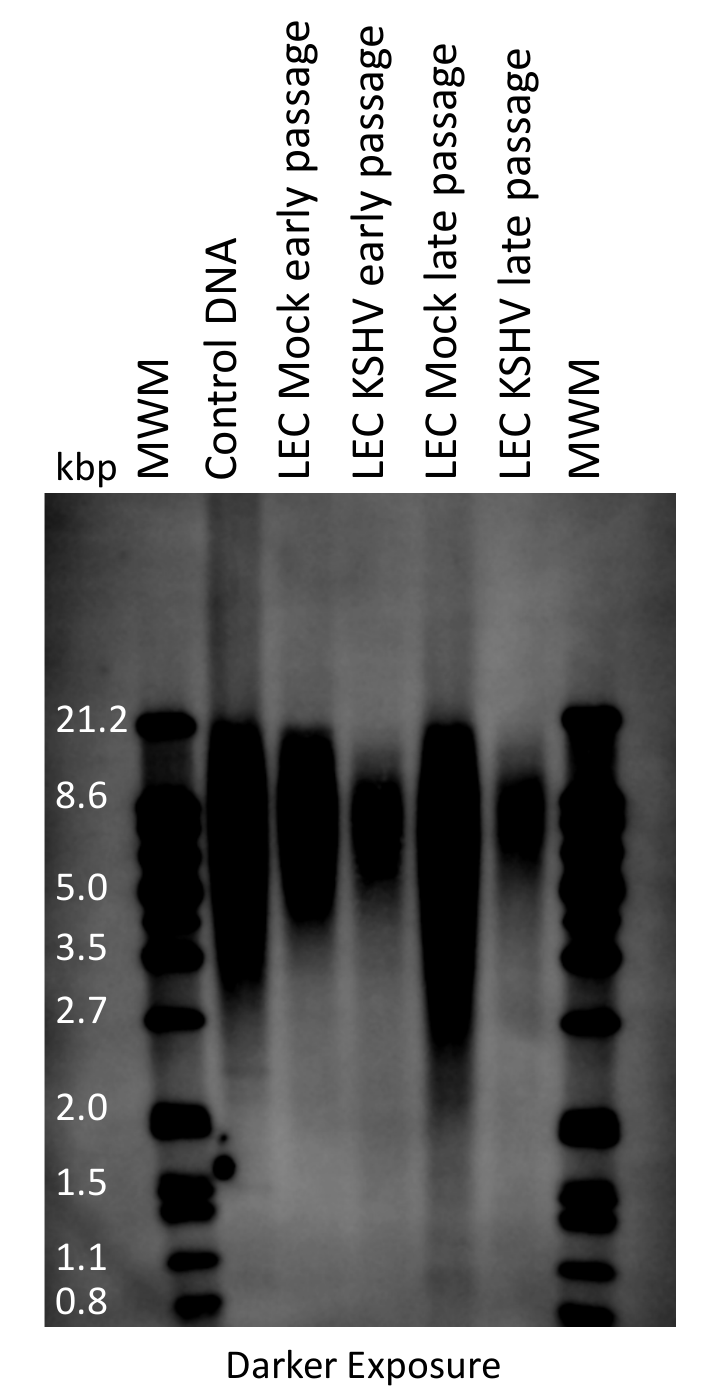

Supplement: S1 Fig — Southern blot of total DNA from early and late passage mock- and KSHV-infected LECs probed with a telomerase-specific probe. A darker exposure of Fig 4B. (TIF) [file ppat.1008634.s001.tif]

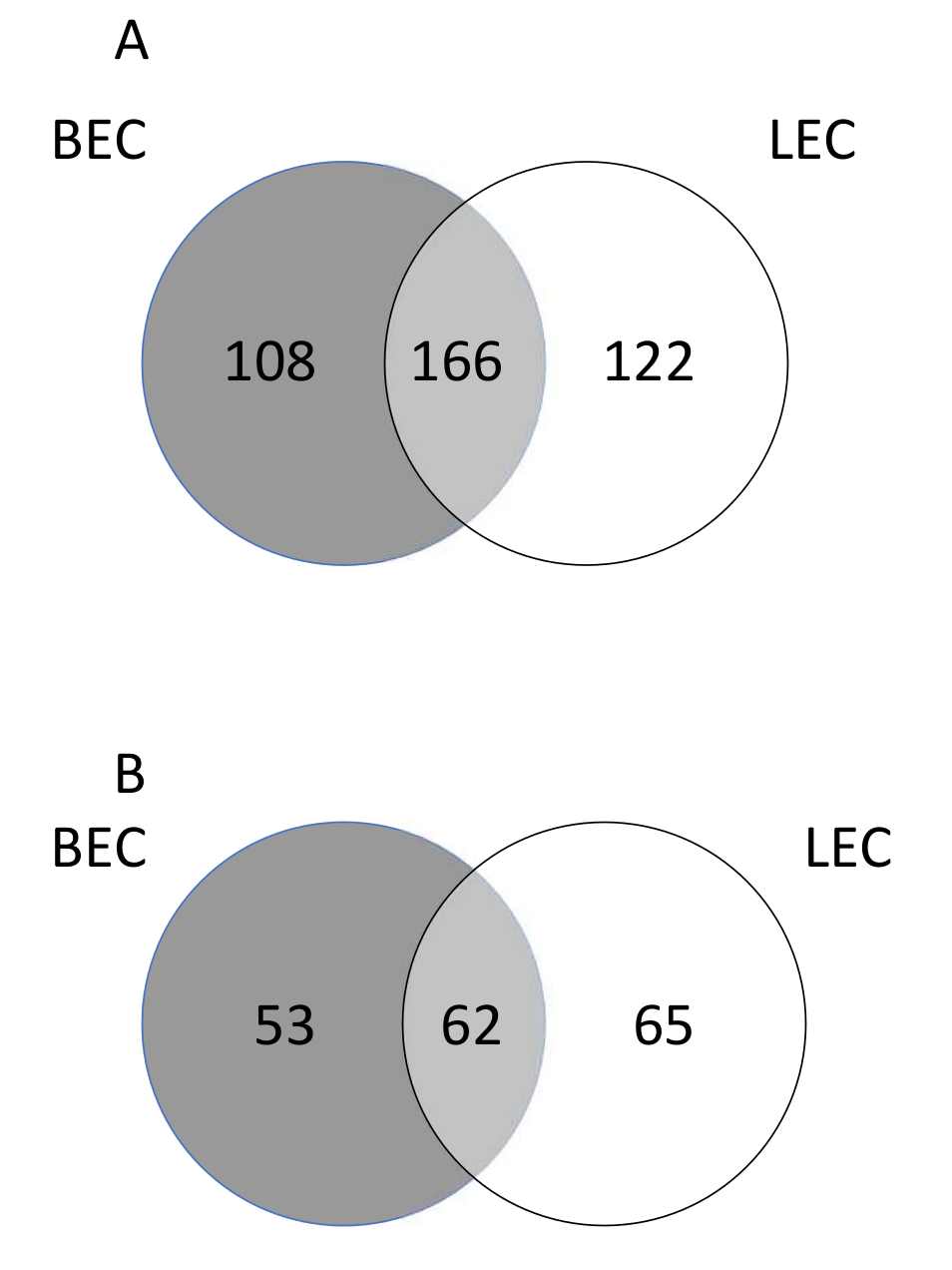

Supplement: S2 Fig — (A) Venn diagram showing genes that are upregulated by KSHV in BECs (dark circle) and LECs (light circle). (B) Venn diagram showing genes that are downregulated by KSHV in BECs (dark circle) and LECs (light circle). (TIF) [file ppat.1008634.s002.tif]
