# Supplementary material for: KSHV requires vCyclin to overcome replicative senescence in primary human lymphatic endothelial cells
Source: PLoS Pathog. 2020 Jun 18;16(6):e1008634. doi: 10.1371/journal.ppat.1008634 (PMC7326280; doi:10.1371/journal.ppat.1008634)
Supplement: S1 Table — (DOCX) [file ppat.1008634.s003.docx]

**S1 Table. Expression data for blood and lymphatic endothelial cell markers in different cell isolates.**

| Normalized Ct value | BEC1 | BEC2 | BEC3 | LEC1 | LEC2 | LEC3 |
| --- | --- | --- | --- | --- | --- | --- |
| VEGFR1 | 6.12 | 13.01 | 7.04 | 6.645 | 9.075 | 8.22 |
| VEGFR3 | 7.67 | 7.88 | 6.95 | 6.92 | 5.775 | 6.68 |
| Prox-1 | 12.18 | 19.46 | 11.10 | 4.49 | 6.21 | 6.64 |
