# Supplementary material for: KSHV requires vCyclin to overcome replicative senescence in primary human lymphatic endothelial cells
Source: PLoS Pathog. 2020 Jun 18;16(6):e1008634. doi: 10.1371/journal.ppat.1008634 (PMC7326280; doi:10.1371/journal.ppat.1008634)
Supplement: S2 Table — (DOCX) [file ppat.1008634.s004.docx]

**S2 Table. Pathways upregulated by WT KSHV but not ΔvCyclin.**

| Gene Set Name | # Genes in Gene Set (K) | # Genes in Overlap (k) | k/K | p-value | FDR q-value |
| --- | --- | --- | --- | --- | --- |
| HALLMARK_ESTROGEN_RESPONSE_LATE | 200 | 19 | 0.095 | 8.05E-13 | 4.03E-11 |
| HALLMARK_MYOGENESIS | 200 | 18 | 0.09 | 8.06E-12 | 1.77E-10 |
| HALLMARK_IL6_JAK_STAT3_SIGNALING | 87 | 13 | 0.1494 | 1.06E-11 | 1.77E-10 |
| HALLMARK_COAGULATION | 138 | 15 | 0.1087 | 3.03E-11 | 3.79E-10 |
| HALLMARK_COMPLEMENT | 200 | 16 | 0.08 | 6.72E-10 | 4.80E-09 |
| HALLMARK_EPITHELIAL_MESENCHYMAL_TRANSITION | 200 | 16 | 0.08 | 6.72E-10 | 4.80E-09 |
| HALLMARK_KRAS_SIGNALING_UP | 200 | 16 | 0.08 | 6.72E-10 | 4.80E-09 |
