# Supplementary material for: KSHV requires vCyclin to overcome replicative senescence in primary human lymphatic endothelial cells
Source: PLoS Pathog. 2020 Jun 18;16(6):e1008634. doi: 10.1371/journal.ppat.1008634 (PMC7326280; doi:10.1371/journal.ppat.1008634)
Supplement: S3 Table — (DOCX) [file ppat.1008634.s005.docx]

**S3 Table.** **Pathways downregulated by WT KSHV but not ΔvCyclin.**

| Gene Set Name | # Genes in Gene Set (K) | # Genes in Overlap (k) | k/K | p-value | FDR q-value |
| --- | --- | --- | --- | --- | --- |
| HALLMARK_TNFA_SIGNALING_VIA_NFKB | 200 | 42 | 0.21 | 2.58E-42 | 1.29E-40 |
| HALLMARK_EPITHELIAL_MESENCHYMAL_TRANSITION | 200 | 25 | 0.125 | 7.69E-20 | 1.92E-18 |
| HALLMARK_KRAS_SIGNALING_UP | 200 | 21 | 0.105 | 2.53E-15 | 4.22E-14 |
| HALLMARK_APOPTOSIS | 161 | 18 | 0.1118 | 8.41E-14 | 1.05E-12 |
| HALLMARK_APICAL_JUNCTION | 200 | 19 | 0.095 | 3.35E-13 | 2.79E-12 |
| HALLMARK_INFLAMMATORY_RESPONSE | 200 | 19 | 0.095 | 3.35E-13 | 2.79E-12 |
| HALLMARK_UV_RESPONSE_DN | 144 | 16 | 0.1111 | 2.24E-12 | 1.60E-11 |
| HALLMARK_ESTROGEN_RESPONSE_EARLY | 200 | 14 | 0.07 | 2.31E-08 | 1.28E-07 |
| HALLMARK_P53_PATHWAY | 200 | 14 | 0.07 | 2.31E-08 | 1.28E-07 |
| HALLMARK_IL2_STAT5_SIGNALING | 200 | 13 | 0.065 | 1.73E-07 | 8.67E-07 |
