# Supplementary material for: KSHV requires vCyclin to overcome replicative senescence in primary human lymphatic endothelial cells
Source: PLoS Pathog. 2020 Jun 18;16(6):e1008634. doi: 10.1371/journal.ppat.1008634 (PMC7326280; doi:10.1371/journal.ppat.1008634)
Supplement: S1 Text — (DOCX) [file ppat.1008634.s006.docx]

**Supplemental Materials and Methods**

**RNA-sequencing**

Total RNA was isolated from mock- and KSHV-infected HMVECs at 48 hours post infection, or KSHV- or ΔvCyclin-infected LECs at 1 week post infection using the NucleoSpin RNA kit (Machery-Nagle, Bethlehem, PA). Samples were isolated from 3 separate infection replicates. RNA was further concentrated and purified using the RNA Clean and Concentrator kit (Zymo Research, Irvine, CA). Purified RNA samples were processed at the Fred Hutchison Cancer Research Center Genomic Resources core facility and sequenced using an Illumina HiSeq 2000. Image analysis and base calling were performed using RTA v1.17 software (Illumina). Reads were aligned to the Ensembl's GRCh37 release 70 reference genome using TopHat v2.08b and Bowtie 1.0.0 [1,2] Counts for each gene were generated using htseq-count v0.5.3p9. Differentially expressed genes were determined using the R package EdgeR (Bioconductor). Genes were called significant with a |logFC| > 0.585 and a false discovery rate of <0.05. Gene Ontology enrichment was performed using Cytoscape and BINGO [3].

Gene Set Enrichment was performed using the Molecular Signatures Database at [http://software.broadinstitute.org/gsea/index.jsp [](http://software.broadinstitute.org/gsea/index.jsp%20%5b)4,5]. Additionally, the data discussed in this publication have been deposited in NCBI's Gene Expression Omnibus [6] and are accessible through GEO Series accession number GSE54416 (<http://www.ncbi.nlm.nih.gov/geo/query/acc.cgi?acc=GSE54416)> and GEO Series accession number GSE136654 (<https://www.ncbi.nlm.nih.gov/geo/query/acc.cgi?acc=GSE136654>).

1. Trapnell C, Pachter L, Salzberg SL. TopHat: discovering splice junctions with RNA-Seq. *Bioinformatics*. 2009;25(9):1105-11.
2. Langmead B, Trapnell C, Pop M, Salzberg SL. Ultrafast and memory-efficient alignment of short DNA sequences to the human genome. *Genome Biol*. 2009;10(3):R25
3. Saito R, Smoot ME, Ono K, Ruscheinski J, Wang PL, Lotia S, et al. A travel guide to Cytoscape plugins. *Nat Methods*. 2012;9(11):1069-76.
4. Subramanian A, Tamayo P, Mootha VK, Mukherjee S, Ebert BL, Gillette MA, et al. Gene set enrichment analysis: a knowledge-based approach for interpreting genome-wide expression profiles. Proc Natl Acad Sci U S A. 2005 Oct 25;102(43):15545-50. Epub 2005 Sep 30.
5. Mootha VK, Lindgren CM, Eriksson KF, Subramanian A, Sihag S, Lehar J, et al. PGC-1alpha-responsive genes involved in oxidative phosphorylation are coordinately downregulated in human diabetes. Nat Genet. 2003 Jul;34(3):267-73.
6. Edgar R, Domrachev M, Lash AE. Gene Expression Omnibus: NCBI gene expression and hybridization array data repository. *Nucleic Acids Res*. 2002;30(1):207-10.
